# The mediating and moderating roles of surface acting and perceived organizational support in the relationship between perceived overqualification and silence behavior among young nurses: A cross-sectional study

**DOI:** 10.1371/journal.pone.0356282

**Published:** 2026-08-13

**Authors:** Dongrun Liu, Qixu Zhou, Shiwei Ma, Song Zhang, Yang Liu, Huanhuan Zhang, Kaige Li, Chaoran Chen

**Affiliations:** 1 Institute of Nursing and Health, School of Nursing and Health, Henan University, Kaifeng, China; 2 Huaihe Hospital, Henan University, Kaifeng, China; 3 School of Nursing and Health, Zhengzhou University, Zhengzhou, Henan, China; Universiti Sains Malaysia - Kampus Kesihatan, MALAYSIA

## Abstract

**Purpose:**

Young nurses represent the primary workforce for the future of the nursing industry. However, they often experience a heightened sense of overqualification, which can easily lead to silence behavior, negatively impacting personal well-being, organizational development, and the quality of nursing care. The purpose of this study is to explore the relationship between perceived overqualification and silence behavior among young nurses, as well as the mediating role of surface acting and the moderating role of perceived organizational support.

**Method:**

The Scale of Perceived Overqualification, the surface acting dimension of the Emotional Labor Scale, the Employee Silence Scale, and the Perceived Organizational Support Scale were used to conduct a cross-sectional survey of 1,095 young nurses from six general hospitals in China. A moderated mediation model was constructed, and data were analyzed using Hayes’ PROCESS macro (Models 4 and 59).

**Results:**

There are significant correlations among perceived overqualification, surface acting, silence behavior, and perceived organizational support (p < 0.01). The direct effect of perceived overqualification on silence behavior was significant (Effect = 0.081, 95% CI = [0.024, 0.138]), and the mediating effect of surface acting on the relationship between perceived overqualification and silence behavior was also significant (Effect = 0.111, 95% CI = [0.084, 0.142]). Furthermore, perceived organizational support played a negative moderating role in the relationships among perceived overqualification, surface acting, and silence behavior. Specifically, when perceived organizational support was high, both the direct and indirect effects of perceived overqualification on silence behavior were weakened and even showed a tendency to reverse direction.

**Conclusion:**

Reducing young nurses’ perceived overqualification, decreasing surface acting, and enhancing perceived organizational support may help reduce the occurrence of silence behavior. Nursing managers should provide young nurses with appropriate career development opportunities, offer training in emotional regulation and stress management, and foster an open, psychologically safe communication environment and supportive organizational climate. These strategies may effectively intervene in silence behavior and contribute to improving team vitality and the quality of nursing services.

## 1. Introduction

Nurses play a crucial role in healthcare systems, bearing significant responsibilities in health promotion, prevention, treatment, and rehabilitation [1,2]. Young nurses, defined as those aged 18–35, are increasingly entering clinical nursing as a result of the development of higher nursing education in recent years, contributing new vitality to the sustainable development of the nursing profession [3,4]. A noteworthy issue is that young nurses are more likely to experience perceived overqualification compared to older nurses [4,5]. Perceived overqualification refers to the feeling that one’s education, experience, and skills exceed the requirements of the job [6]. Research shows that at least one-third of employees globally feel overqualified for their positions, with nurses reporting higher levels of perceived overqualification than other professions [7]. Perceived overqualification can negatively affect nurses’ career development, physical and mental health, subjective well-being, work attitudes, and behavior, ultimately damaging care quality [7,8]. Due to unrealistic expectations of their work, young nurses often feel that their value and strengths are underutilized, leading to perceived overqualification and a series of adverse work behaviors [4,8,9]. This presents challenges for hospital management and warrants broader attention.

Based on Hobfoll’s Conservation of Resources Theory [10], perceived overqualification represents a resource loss: when employees feel overqualified, they believe they are expending resources on a job that does not align with their abilities and developmental needs, without gaining additional resources. This triggers dissatisfaction, alienation, and decreased work motivation [7]. To avoid further resource depletion, employees may adopt defensive strategies, such as silence behavior [11]. Silence behavior refers to the intentional withholding of important information, suggestions, ideas, or concerns from work or the organization [12]. Silence behavior is common in nursing [13]. Yurdakul et al. [14] found that over 90% of nurses reported engaging in silence behavior at work, with 61.6% choosing to remain silent even during critical moments, which is especially evident among young nurses. However, extensive research shows that silence behavior impedes communication within nursing teams, reduces nurse performance, conceals patient safety risks, negatively affects care quality, causes loss of critical information, and hinders hospital efficiency and innovation [15–17]. This harms both young nurses and the organization, impacting care quality and patient safety. Liu et al. [18] also found a positive correlation between perceived overqualification and silence behavior. According to relative deprivation theory, when employees expect social status, compensation, benefits, and interpersonal relationships from their work, overqualified individuals feel that the job does not meet their expectations, creating a sense of deprivation [6,19]. Perceived overqualification may trigger dissatisfaction and shame in young nurses, increasing their tendency to withdraw and avoid, thus driving them to use silence behavior to escape this discomfort [18,20]. However, no studies have yet explored the impact of perceived overqualification on silence behavior among young nurses. Given that young nurses are increasingly becoming the backbone of the nursing profession, this study aims to explore the relationship between perceived overqualification and silence behavior from their perspective. The goal is to provide a theoretical basis for improving young nurses’ silence behavior, which has significant implications for both the nurses themselves, organizations, and patients. Therefore, based on Conservation of Resources theory and relative deprivation theory, we proposed that the negative emotions and avoidance tendencies induced by perceived overqualification may serve as important psychological triggers of silence behavior. Accordingly, we proposed Hypothesis 1: Perceived overqualification among young nurses positively predicts silence behavior.

In addition to cognitive and physical labor, a new form of labor called emotional labor has emerged. Emotional labor is the process of adjusting one’s emotions, feelings, and behaviors to achieve organizational goals [21]. According to the three-level theory of emotional labor [22], emotional labor can be categorized into three levels: natural expressions, deep acting, and surface acting. Surface acting involves modifying external visible behaviors to exhibit the required emotions in a work context, while internal feelings remain unchanged. Hu et al. [23] found that perceived overqualification positively predicts surface acting in emotional labor. Young nurses with higher perceived overqualification may feel that their expectations of the organization have not been met [24], leading to a lack of recognition for their profession and diminished work passion [5]. As a result, they tend to engage in surface acting, where their outward emotional expression does not align with their true feelings [23]. Additionally, Xie [25] found that surface acting positively influences silence behavior. Consistently masking emotions while disregarding internal feelings leads to emotional exhaustion and impacts work behavior and state, further increasing silence behavior [11,26]. Therefore, surface acting may serve as an important mediating mechanism between perceived overqualification and silence behavior. Based on this assumption, we proposed Hypothesis 2: Surface acting mediates the relationship between perceived overqualification and silence behavior.

Perceived organizational support refers to the recognition of employees’ contributions and the organization’s concern for their welfare [27]. It enables nurses to feel cared for by the organization, enhancing their sense of belonging and professional identity, which motivates them to work more actively and provide better care to patients [28]. Studies have shown that perceived organizational support moderates the relationship between nurses’ negative emotions and work behavior [29,30]. Furthermore, Yao et al. [31] found that perceived organizational support can mitigate the negative effects of perceived overqualification on nurses. Perceived organizational support may make young nurses feel valued by the organization, reducing emotional exhaustion caused by perceived overqualification, boosting self-confidence, and encouraging them to proactively contribute to the organization, rather than remain silent [32]. Xu et al. [33] also showed that perceived organizational support influences surface acting in emotional labor. According to social comparison theory, when nurses perceive higher organizational support, it alleviates the negative emotions associated with perceived overqualification, reduces feelings of deprivation and unfairness, and enhances their enthusiasm, self-identity, and achievement, which in turn reduces surface acting [5,31,34]. Nurses who perceive high organizational support are also more likely to adjust their true emotions and behaviors in response to work demands, rather than engage in emotional masking. They tend to exhibit positive work behaviors to reciprocate the support and care from the organization, thus reducing silence behavior [35,36]. Therefore, we hypothesized that perceived organizational support plays an important moderating role by suppressing the negative emotional and behavioral responses triggered by perceived overqualification. Specifically, when young nurses perceive higher organizational support, the relationships between perceived overqualification and surface acting, perceived overqualification and silence behavior, as well as surface acting and silence behavior, may be weakened. Accordingly, the following hypotheses were proposed:

Hypothesis 3: Perceived organizational support negatively moderates the relationship between perceived overqualification and silence behavior.

Hypothesis 4: Perceived organizational support negatively moderates the relationship between perceived overqualification and surface acting.

Hypothesis 5: Perceived organizational support negatively moderates the relationship between surface acting and silence behavior.

**Fig 1:**
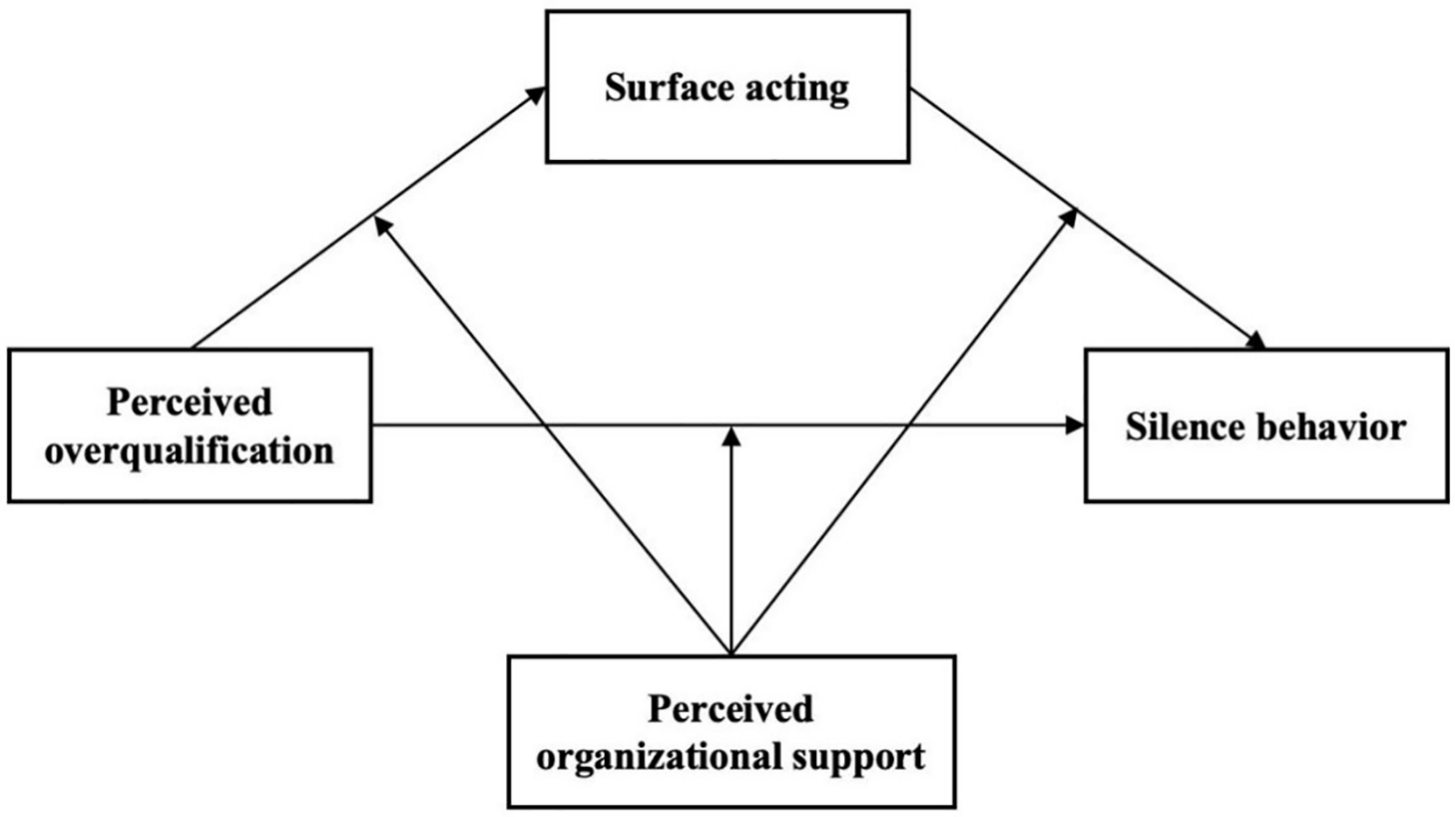
Conceptual model.

In conclusion, based on Conservation of Resources Theory, this study explores the relationship between perceived overqualification and silence behavior among young nurses and constructs a mediating-moderating model. This model aims to provide a comprehensive understanding of how perceived overqualification affects silence behavior, with surface acting as a mediator and perceived organizational support as a moderator. The research findings provide a theoretical basis for reducing nurses’ silence behaviors caused by perceived overqualification and surface acting, which contributes to optimizing nursing management strategies and promoting the long-term sustainable development of organizations. The conceptual model of this study is shown in Fig 1.

## 2. Materials and methods

### 2.1. Design

This study is a cross-sectional investigation conducted in accordance with the STROBE guidelines to ensure transparency and reporting quality.

### 2.2. Sample and data collection

A multistage sampling strategy was employed in this study. During the hospital selection stage, six general hospitals located in central China were selected using convenience sampling between November and December 2024. These hospitals were considered representative in terms of regional economic development and healthcare resource allocation. Within the selected hospitals, a stratified cluster sampling method was adopted. Specifically, hospital departments were first stratified according to department categories. Then, departments were randomly selected as sampling clusters from each stratum, and all eligible young nurses within the selected departments were invited to participate in the questionnaire survey. This approach was used to enhance the representativeness and diversity of the sample.

The survey did not collect participants’ names or any identifiable information. The research team coordinated with hospital human resources departments to ensure cooperation and provided participants with private spaces, ample time, and assurances that the data would be used only for this research, ensuring the anonymity, confidentiality, and validity of the data.

Inclusion criteria were: (1) age between 18 and 35 years; (2) clinically registered nurse; (3) informed consent and voluntary participation in the survey. Exclusion criteria were: (1) nurses not directly involved in patient care and (2) student nurses.

The required sample size was calculated using the formula N = 4Uα^2^S^2^/δ^2^ [37], which is widely applied for sample size estimation in cross-sectional studies in the nursing field. In this formula, Nrepresents the minimum required sample size, Uα represents the critical value of the standard normal distribution corresponding to the significance level α, S represents the standard deviation of the primary outcome variable obtained from the preliminary survey, and δ represents the allowable error. Before the formal investigation, a pilot study was conducted among 40 young clinical nurses who met the inclusion criteria but were not included in the formal survey. The same measurement instruments and survey procedures as those used in the formal study were adopted. Based on the pilot study results, descriptive statistical analysis was performed on the mean item score of the primary outcome variable, silence behavior. The standard deviation was calculated as S = 1.51, which was subsequently used for sample size estimation. With α = 0.05 (Uα = 1.96) and δ = 0.20, the calculated minimum required sample size was approximately 876 participants. Considering potential invalid questionnaires, missing data, and non-response, a total of 1,200 questionnaires were distributed. Invalid questionnaires were excluded based on criteria such as incomplete responses, missing data, abnormal completion times, or patterned answering. After removing 105 invalid questionnaires, 1,095 valid questionnaires were retained, yielding a valid response rate of 91.25%.

### 2.3. Measures

#### 2.3.1. Scale of perceived overqualification (SPOQ).

Perceived overqualification was measured using the SPOQ developed by Maynard et al. [38] and translated by Yang [39]. The scale has been validated for use in Chinese nursing contexts [5]. It consists of 9 items rated on a 6-point Likert scale (1 = strongly disagree, 6 = strongly agree). Higher scores indicate stronger perceived overqualification. In this study, the Cronbach’s α coefficient was 0.913.

#### 2.3.2. Emotional labor scale (ELS).

Surface acting was measured using the surface acting dimension of the ELS developed by Diefendorff et al. [22] and translated by Bai. [40] The scale has demonstrated good applicability in Chinese nursing settings [41]. It contains 7 items rated on a 5-point Likert scale (1 = not at all true, 5 = completely true). Higher scores reflect higher levels of surface acting. In this study, the Cronbach’s α coefficient for this dimension was 0.854.

#### 2.3.3. Employee silence scale (ESS).

Silence behavior was assessed using the ESS developed by Tangirala and Ramanujam [42] and revised by Li et al. [43] The scale has strong psychometric properties and is widely used in nursing research [44]. It includes 5 items rated on a 6-point Likert scale (1 = never, 6 = very frequently). Higher scores indicate more frequent silence behavior. In this study, the Cronbach’s α coefficient was 0.883.

#### 2.3.4. Perceived organizational support scale (POSS).

Perceived organizational support was measured using the POSS developed by Chen [45] and adapted by Zuo [46] for Chinese nursing practice. The scale includes 13 items across emotional support and instrumental support dimensions, rated on a 5-point Likert scale (1 = strongly disagree, 5 = strongly agree). Higher scores reflect stronger perceived organizational support. In this study, the Cronbach’s α coefficient was 0.943.

### 2.4. Ethical considerations

This study involved an anonymous survey, did not include unethical practices or human clinical experiments, and posed no physical or psychological risks to participants. Prior to distributing the questionnaires, informed consent was obtained from hospital administrators and nursing departments. Participants were informed of the study’s purpose and significance and all provided written informed consent before participation in the study. They were assured that their responses would be used solely for this research and that they could withdraw at any time. The study was conducted following the Declaration of Helsinki and approved by the Henan University Biological Sciences Research Ethics Committee [Approval Number HUSOM2024−572].

### 2.5. Statistical analysis

Data were analyzed using SPSS 26.0 and PROCESS 4.1. First, Harman’s single-factor test was conducted to assess common method bias, and descriptive statistics were calculated for demographic characteristics. Pearson’s correlation analysis was then used to examine relationships among the four variables. Next, Hayes’ PROCESS macro (Model 4 and Model 59) was applied to test the moderated mediation model. Model 4 was used to examine whether surface acting mediated the relationship between perceived overqualification and silence behavior. Model 59 tested whether perceived organizational support moderated both the direct and indirect paths between perceived overqualification and silence behavior. Finally, mediation and moderated mediation effects were analyzed, and simple slope plots were generated. We adopted 5,000 bootstrapped samples to calculate bias-corrected 95% confidence intervals (CI). All p-values were two-tailed, with p < 0.05 indicating statistical significance.

## 3. Results

### 3.1. Common method bias tests

Harman’s single-factor test was used to examine common method bias. The results showed that the first factor explained 24.58% of the variance, which is less than 40%, indicating that there is no significant common method bias in this study.

### 3.2. Participants’ demographic characteristics

Table 1 presents the demographic characteristics of the participants. Among the 1095 young nurses who participated, 26.21% were male and 73.79% were female, with an age range of 18–35 years; 78.17% were single; 83.93% held a bachelor’s degree or higher; 52.69% were contract employees; 81.73% had a monthly income below 7000 RMB; 62.92% held the title of Nurse; and only 7.21% were head nurses.

**Table 1:** Demographic Characteristics of Participants (*N* = 1095).

Demographics	N (%)
Gender	
Male	287(26.21)
Female	808(73.79)
Age (Year)	
18-24	583(53.24)
25-29	372(33.97)
30-35	140(12.79)
Marital status	
Single	856(78.17)
Married Others^a^	228(20.82) 11(1.01)
Education	
≤Associate’s degree	176(16.07)
Bachelor’s degree ≥Master’s degree	699(63.84) 220(20.09)
Labor and personnel relations	
Formal establishment	216(19.73)
Personnel agency Contract system	302(27.58) 577(52.69)
Monthly income (RMB)	
< 4000	464(42.37)
4000∼7000 7000∼10,000	431(39.36) 125(11.42)
> 10,000	75(6.85)
Professional title	
Nurse	689(62.92)
Senior nurse	261(23.84)
Nurse-in-charge	94(8.58)
Deputy chief nurse or above	51(4.66)
Head nurse?	
Yes	79(7.21)
No	1016(92.79)

^a^Others: divorced and widowed

### 3.3. Pearson’s correlation analysis

Means (M), standard deviations (SD), and Pearson’s correlations for each variable are presented in Table 2. The average item score for perceived overqualification was 3.53 ± 0.86, for surface acting was 3.04 ± 0.91, for silence behavior was 3.37 ± 1.15, and for perceived organizational support was 2.93 ± 0.95. The correlations between all variables reached statistical significance (P < 0.01).

**Table 2:** Descriptive statistics and correlations among the study variables (*N* = 1095).

	M	SD	1	2	3	4
1.PO	3.53	0.86	—			
2.SA	3.04	0.91	0.297^**^	—		
3.SB	3.37	1.15	0.192^**^	0.399^**^	—	
4.POS	2.93	0.95	−0.093^**^	−0.096^**^	−0.101^**^	—

PO, perceived overqualification; SA, surface acting; SB, silence behavior; POS, perceived organizational support

** p < 0.01 (two-tailed)

### 3.4. Moderated mediation model analysis

**Fig 2:**
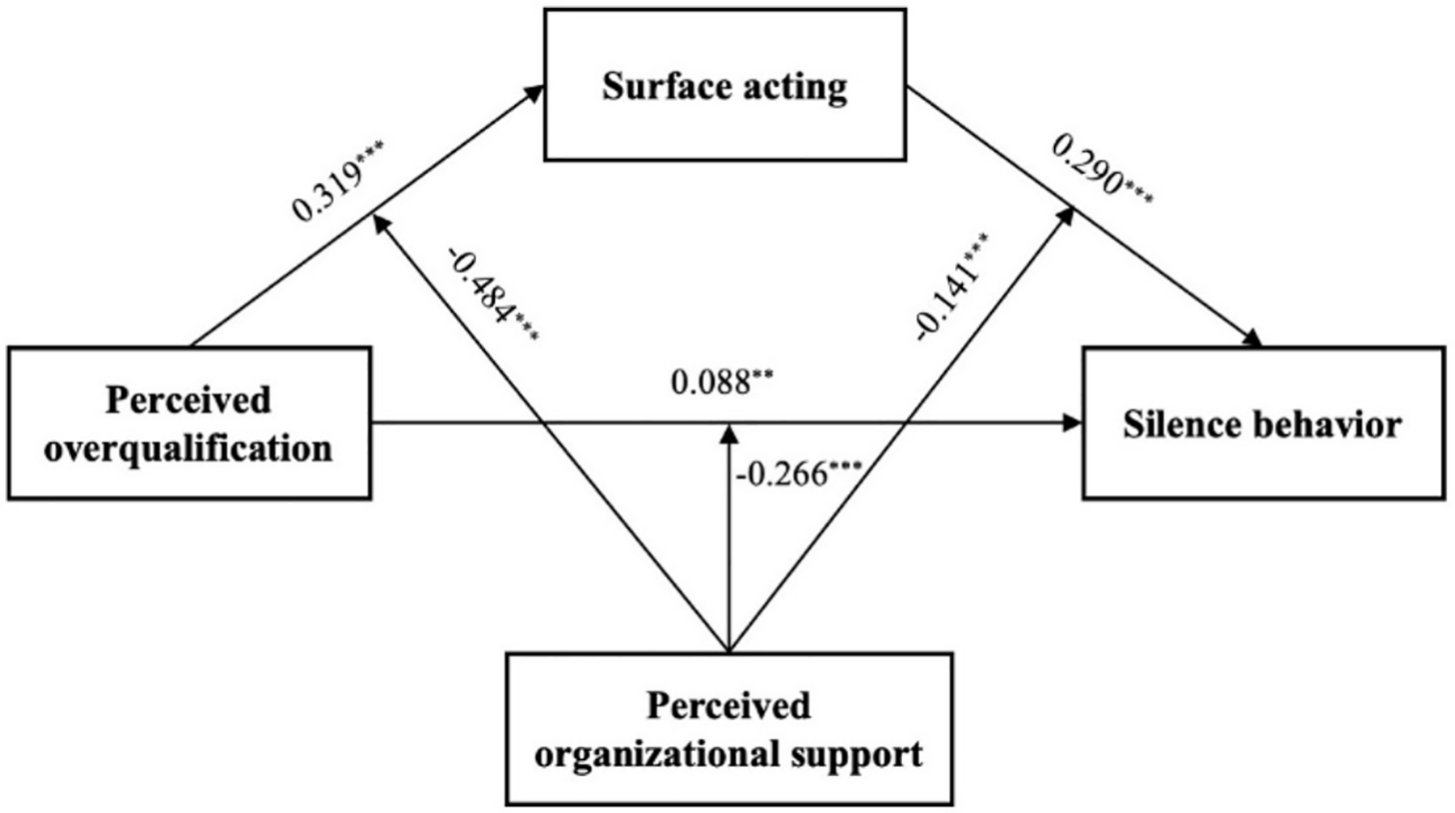
Moderated mediation model.

As shown in Table 3, we used SPSS PROCESS Model 4 and Model 59 to test the moderated mediation model, all data have been standardized. The results revealed: Perceived overqualification positively predicted silence behavior (*β* = 0.192, 95% CI=[0.134, 0.251], *P* < 0.001); Perceived overqualification positively predicted surface acting (*β* = 0.297, 95% CI=[0.240, 0.354], *P* < 0.001); After including surface acting as a mediator, perceived overqualification still positively predicted silence behavior (*β* = 0.081, 95% CI=[0.024, 0.138], *P* < 0.01), while surface acting also positively predicted silence behavior (*β* = 0.375, 95% CI=[0.318, 0.432], *P* < 0.001); After adding perceived organizational support as a moderator, perceived overqualification continued to positively predict surface acting (*β* = 0.319, 95% CI=[0.265, 0.373], *P* < 0.001). The interaction effect between perceived overqualification and perceived organizational support on surface acting was significant (*β* = −0.484, 95% CI=[−0.566, −0.402], *P* < 0.001), indicating that perceived organizational support negatively moderated the relationship between perceived overqualification and surface acting; Perceived overqualification still positively predicted silence behavior (*β* = 0.088, 95% CI=[0.031, 0.145], *P* < 0.01), and surface acting also positively predicted silence behavior (*β* = 0.290, 95% CI=[0.231, 0.349], *P* < 0.001). The interaction effect between perceived overqualification and perceived organizational support on silence behavior was significant (*β* = −0.266, 95% CI=[−0.352, −0.181], *P* < 0.001), indicating that perceived organizational support negatively moderated the relationship between perceived overqualification and silence behavior. Simultaneously, the interaction effect between surface acting and perceived organizational support on silence behavior was significant (*β* = −0.141, 95% CI=[−0.193, −0.089], *P* < 0.001), indicating that perceived organizational support negatively moderated the relationship between surface acting and silence behavior. The moderated mediation model is illustrated in Fig 2.

**Table 3:** Testing the moderated mediation model (*N* = 1095).

Dependent variables	Independent variables	*R*	*R* ^*2*^	*F*	*β*	*SE*	LLCI	ULCI	*P*
SB		0.192	0.037	42.001					
	PO				0.192	0.030	0.134	0.251	<0.001
SA		0.297	0.088	105.792					
	PO				0.297	0.029	0.240	0.354	<0.001
SB		0.406	0.165	107.899					
	PO				0.081	0.029	0.024	0.138	<0.01
	SA				0.375	0.029	0.318	0.432	<0.001
SA		0.439	0.193	86.74					
	PO				0.319	0.027	0.265	0.373	<0.001
	POS				−0.151	0.028	−0.206	−0.096	<0.001
	PO × POS				−0.484	0.042	−0.566	−0.402	<0.001
SB		0.468	0.219	60.99					
	PO				0.088	0.029	0.031	0.145	<0.01
	SA				0.290	0.030	0.231	0.349	<0.001
	POS				−0.150	0.029	−0.207	−0.093	<0.001
	PO × POS				−0.266	0.044	−0.352	−0.181	<0.001
	SA × POS				−0.141	0.027	−0.193	−0.089	<0.001

PO, perceived overqualification; SA, surface acting; SB, silence behavior; POS, perceived organizational support

**Fig 3:**
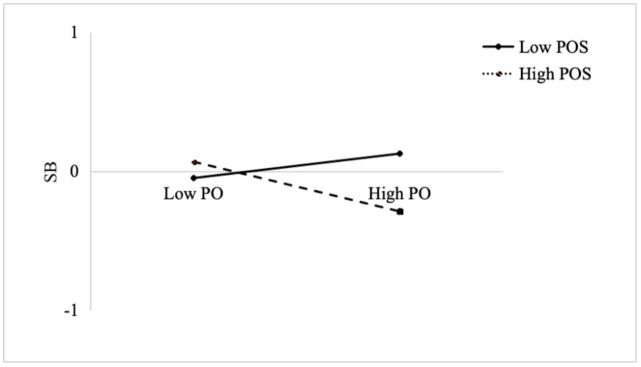
The moderating role of perceived organizational support in the relationship between perceived overqualification and silence behavior.

**Fig 4:**
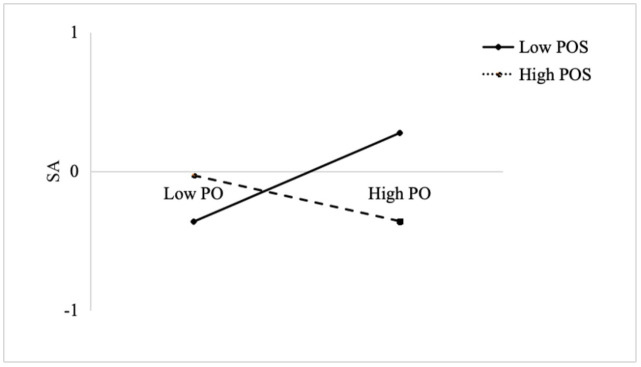
The moderating role of perceived organizational support in the relationship between perceived overqualification and surface acting.

**Fig 5:**
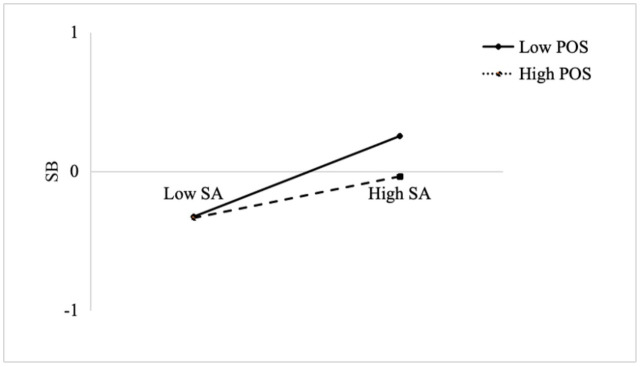
The moderating role of perceived organizational support in the relationship between surface acting and silence behavior.

The results of the simple slope analysis for the interaction effects are shown in Figs 3–5. In this study, the mean ± 1 standard deviation (M ± 1SD) method was used to classify variables into high and low scores. Specifically, values equal to M + 1SD were defined as high scores, whereas values equal to M − 1SD were defined as low scores. The results showed that: (1) The relationship between perceived overqualification and silence behavior was weakened under high perceived organizational support compared to low perceived organizational support, and even turned negative; (2) The relationship between perceived overqualification and surface acting was weakened under high perceived organizational support compared to low perceived organizational support, and even turned negative; (3) The relationship between surface acting and silence behavior was weakened under high perceived organizational support compared to low perceived organizational support.

Furthermore, we used the 5000 resample bootstrapping method with a 95% CI to test the mediation and moderated mediation effects, as detailed in Table 4. The results showed: (1) The direct effect of perceived overqualification on silence behavior was significant (Effect = 0.081, 95% CI = [0.024, 0.138]), indicating that perceived overqualification directly and positively predicted silence behavior, thereby supporting Hypothesis 1. (2) The mediating effect of surface acting between perceived overqualification and silence behavior was also significant (Effect = 0.111, 95% CI = [0.084, 0.142]), indicating that surface acting mediated the relationship between perceived overqualification and silence behavior among young nurses, thereby supporting Hypothesis 2. (3) Perceived organizational support negatively moderated the direct relationship between perceived overqualification and silence behavior. At low levels of perceived organizational support, the direct effect was significantly positive (Effect = 0.354, 95% CI = [0.243, 0.466]); At average levels of perceived organizational support, the direct effect remained significantly positive but was reduced in magnitude (Effect = 0.088, 95% CI = [0.031, 0.145]); At high levels of perceived organizational support, the direct effect was significantly negative (Effect = −0.179, 95% CI = [−0.272, −0.086]). These findings supported Hypothesis 3. (4) Perceived organizational support negatively moderated the indirect effect of perceived overqualification on silence behavior through surface acting. At low levels of perceived organizational support, the indirect effect was significantly positive (Effect = 0.346, 95% CI = [0.275, 0.428]); At average levels of perceived organizational support, the indirect effect remained significantly positive but was reduced in magnitude (Effect = 0.093, 95% CI = [0.068, 0.121]); At high levels of perceived organizational support, the indirect effect was significantly negative (Effect = −0.025, 95% CI = [−0.050, −0.006]). These findings supported Hypotheses 4 and 5. In summary, perceived organizational support negatively moderated both the direct and indirect effects of perceived overqualification on silence behavior.

**Table 4:** Mediation and moderated mediation tests (*N* = 1095).

Effects	Moderator level (POS)	Effect	*SE*	LLCI	ULCI
Direct effect (PO → SB)		0.081	0.029	0.024	0.138
Indirect effect (PO → SA → SB)		0.111	0.015	0.084	0.142
Moderated direct effect (PO → SB)	Low	0.354	0.057	0.243	0.466
	Moderate	0.088	0.029	0.031	0.145
	High	−0.179	0.047	−0.272	−0.086
Moderated indirect effect (PO → SA → SB)	Low	0.346	0.039	0.275	0.428
	Moderate	0.093	0.013	0.068	0.121
	High	−0.025	0.011	−0.050	−0.006

PO, perceived overqualification; SA, surface acting; SB, silence behavior; POS, perceived organizational support

## 4. Discussion

The mean score of silence behavior among participants in this study was at a moderate level, which is consistent with previous studies on nurses [47,48]. Silence behavior is common in nursing work, and its causes are complex [13]. Due to the influence of hierarchical organizational cultures, young nurses generally occupy relatively lower positions within organizational structures. They may worry that expressing their opinions could lead to negative consequences, such as ineffective communication, dissatisfaction from supervisors, or rejection from colleagues. Therefore, they may adopt silence behavior as a self-protective strategy and a means of maintaining organizational harmony [44,49]. As a result, young nurses may tend to withhold their thoughts, opinions, or feelings, and may even remain silent when encountering problems or errors rather than actively communicating or seeking solutions [18,32]. However, the present study used self-reported measures, which may have been influenced by participants’ current emotional states, cognitive evaluations, and other subjective factors. As silence behavior is a potentially risky behavior within organizations, its measurement through self-report methods is particularly susceptible to social desirability bias [39]. Therefore, young nurses may have underestimated their actual level of silence behavior in order to maintain a positive self-image during the survey. Overall, silence behavior is relatively common among young nurses, and it not only affects individuals and organizations but also directly impacts patient safety, leading to more serious consequences. This issue requires sufficient attention and active measures to reduce the occurrence of silence behavior.

The findings of this study demonstrated that perceived overqualification among young nurses was associated with silence behavior, and the direct effect of perceived overqualification on silence behavior was significant. Perceived overqualification may increase negative emotions and attitudes toward nursing work, reduce job satisfaction, and impair work performance [50]. According to Conservation of Resources theory, silence behavior may emerge during this process. Nemati et al. [51] found that nurses are more likely to engage in silence when they perceive unfairness or injustice in the organization, which supports our viewpoint. Young nurses with high perceived overqualification tend to feel that their work does not match their abilities, leading to feelings of deprivation and unfairness, making them more likely to remain silent when problems arise [6,52]. This reduces work efficiency and nursing quality within the team, resulting in numerous harms [50]. However, due to the limitations of the cross-sectional design and self-reported measurement approach, perceived overqualification among young nurses may not fully represent the actual discrepancy between their abilities and job requirements. Therefore, the findings may also be influenced by factors such as individual expectations, reference groups, and career development stages. Further, causal relationships between perceived overqualification and silence behavior cannot be established based on the current findings.

The results also indicated that surface acting among young nurses was associated with both perceived overqualification and silence behavior, and the mediating effect of surface acting on the relationship between perceived overqualification and silence behavior was significant. Surface acting is a superficial form of emotional regulation where employees adjust their facial expressions, body language, and other external cues to meet organizational or professional emotional display requirements, without changing their inner thoughts or feelings [22]. Our findings revealed that young nurses with higher perceived overqualification were also more likely to engage in surface acting at work. They display compliance on the surface but do not genuinely agree with or feel satisfied internally, which leads to a continuous depletion of psychological resources. According to the resource depletion spiral theory [53], this ongoing emotional masking through surface acting results in emotional exhaustion, eventually prompting young nurses to adopt silence as a way to avoid further resource loss [25,54]. This finding suggests that interventions to reduce silence behavior should not only address perceived overqualification at its source but also focus on managing emotional labor, particularly reducing the psychological costs associated with surface acting.

Our study also found that perceived organizational support negatively moderates the relationships between perceived overqualification and silence behavior, perceived overqualification and surface acting, and surface acting and silence behavior. Perceived organizational support negatively moderates both the direct and indirect effects of perceived overqualification on silence behavior. Strong perceived organizational support could directly weaken the association between perceived overqualification and silence behavior among young nurses. Furthermore, high levels of perceived organizational support could alleviate silence behavior associated with surface acting and further reduce the negative emotions caused by perceived overqualification [55], thereby decreasing emotional masking during nursing work and ultimately weakening silence behavior [56]. Previous studies have shown that perceived organizational support can reduce nurses’ negative emotions and improve job enthusiasm and job satisfaction [57]. Research by Song et al. [58] also supports our viewpoint, indicating that a supportive organizational atmosphere and environment have a positive impact on young nurses’ job performance.

Furthermore, we found that as perceived organizational support increases, the direct and indirect effects of perceived overqualification on silence behavior are weakened, even reversing from a significant positive effect to a significant negative effect. Perceived overqualification is a double-edged sword—it can lead to negative effects but also bring some benefits [59]. According to social comparison process theory, when organizations provide sufficient emotional and resource support, they may alter young nurses’ perceptions and coping strategies regarding perceived overqualification. Nurses who possess higher abilities but perceive a mismatch between their qualifications and job requirements may become more likely to utilize their strengths and capabilities [5,31]. High levels of organizational support make young nurses feel valued and respected, and even if they feel overqualified, they are more willing to express their views and talents. This can motivate them to change their situation, such as seeking promotions, raises, or participating in nursing management, and reduce emotional masking in order to fully engage in clinical work. This increases work enthusiasm, leads to more positive work behaviors, and reduces silence behavior [5,59,60]. Therefore, the negative moderating effects observed in this study are theoretically plausible. These findings provide new theoretical support for optimizing nursing management and enhance our understanding of the influence of organizational support. Previous research has primarily focused on the negative consequences of perceived overqualification; however, this study demonstrates that perceived organizational support serves as a critical contextual resource. In highly supportive organizational environments, the positive association between perceived overqualification and silence behavior may be weakened and may even exhibit an opposite pattern, providing theoretical insights and practical guidance for reducing silence behavior.

### 4.1. Implications and recommendations

This study offers the following implications for nursing management practice: First, nursing managers should identify high-potential young nurses and provide them with appropriate development opportunities [61], such as joining research teams, participating in nursing innovation projects, taking on clinical teaching roles, or engaging in nursing decision-making and management. This allows them to leverage their abilities and strengths, helps them see career prospects, and meets their internal needs. Additionally, nursing managers should establish a positive feedback mechanism to alleviate their perception of overqualification. Second, hospitals and nursing managers should provide training in emotional management and stress coping strategies for young nurses to help them master emotional regulation techniques, recognize emotional depletion points, and express their true feelings. This will reduce reliance on surface acting and lower emotional exhaustion [23]. Nursing managers should also create an open, free, and safe communication environment [62], establish “non-punitive error reporting” and “suggestion reward” systems at the department level, and encourage young nurses to voice improvement suggestions and express their true thoughts without fearing negative consequences. Furthermore, nursing managers and hospitals should work together to build a positive organizational culture centered on care, trust, support, and encouragement, thereby improving nurses’ perceptions of organizational support. This will help them feel recognized by the organization and find meaning in their work, increasing work engagement, reducing silence behavior, and promoting active expression [59]. In conclusion, by adopting a “capacity release-emotional regulation-organizational support” triple intervention strategy, the occurrence of silence behavior among young nurses can be reduced, contributing to the improvement of overall nursing quality and the sustainable development of both nursing teams and the organization.

### 4.2. Limitations

This study has several limitations. First, this study adopted a cross-sectional design and collected data only through self-reported measures, which limits the ability to determine the temporal sequence and causal relationships among variables. Additionally, the findings may have been affected by potential social desirability bias. Future studies should employ longitudinal designs combined with objective assessment indicators to further explore the causal relationships and underlying mechanisms among the variables. Second, due to the limitations of the cross-sectional design and the relatively small effect sizes of some moderating effects, the observed moderating effects, although statistically significant, may reflect statistical artifacts rather than genuine reversals of relationships, and their practical significance may therefore be limited. Further validation through future research and practical applications is required. Therefore, the findings should not be simply interpreted as indicating that perceived organizational support inevitably causes perceived overqualification to reduce silence behavior. Instead, they should be understood as suggesting that when perceived organizational support is high, both the direct and indirect effects of perceived overqualification on silence behavior are weakened and may demonstrate an opposite trend. Third, this study focused only on young nurses in central China, resulting in limited geographical representativeness. Moreover, the sample was skewed toward female and unmarried nurses, which may restrict the generalizability of the findings. Future studies should expand to healthcare institutions in different regions and include more diverse samples, particularly male and married nurses, to enhance the external validity and generalizability of the results. Fourth, demographic variables were not included as control variables in the analysis, which may have introduced potential bias due to omitted variables. Future research should incorporate demographic characteristics as control variables to examine differences among young nurses with different personal characteristics and minimize the influence of potential confounding factors.

## 5. Conclusions

This study developed a moderated mediation model and demonstrated that perceived overqualification, surface acting, silence behavior, and perceived organizational support were significantly associated with each other. The direct effect of perceived overqualification on silence behavior was significant, and the mediating effect of surface acting on the relationship between perceived overqualification and silence behavior was also significant. Perceived organizational support played a negative moderating role in the relationships among perceived overqualification, surface acting, and silence behavior. Specifically, when perceived organizational support was high, both the direct and indirect effects of perceived overqualification on silence behavior were weakened and may even show a reversed trend. These findings provide theoretical support and practical implications for optimizing nursing management strategies and reducing silence behavior among young nurses. Nursing managers may reduce silence behavior among young nurses by alleviating perceived overqualification, reducing surface acting, and enhancing perceived organizational support.

## Supporting information

S1 File Dataset used for the study. (XLSX)
